# Central Ohio Dental Society

**Published:** 1865-08

**Authors:** 


### CENTRAL OHIO DENTAL SOCIETY.

By a notice on another page, it will be seen that a society bearing this name is soon to be organized by the profession in central Ohio. This is a movement we have long desired to see consummated. A similar movement was made some four or five years ago, but owing to unauspicious times, failed. Now that prosperity reigns, and from the interest manifested in this matter, we feel assured that a good active society will be formed, one that will result in great good to its members, and an honor to the profession; and we trust that no one in that part of the State, who is worthy of a name and a place in the profession, will withhold his support, but that all will enter heartily and earnestly in the work, and thereby secure to themselves the great benefit deduceable from such instrumentalities.

